# Inhibition of class IIa HDACs reduces mutant HTT aggregation by affecting RNA stability

**DOI:** 10.3389/fnmol.2025.1579194

**Published:** 2025-06-18

**Authors:** Annika Reisbitzer, Cecilia Hollitzer, Adriana Geraci, Jessye Schaefer, Maximilian Burghaus, Jonas Bruns, Joachim Urban, Thomas Kurz, Sybille Krauß

**Affiliations:** ^1^Institute of Biology, Human Biology/Neurobiology, University of Siegen, Siegen, Germany; ^2^Institute of Developmental Biology and Neurobiology, Neural Genetics, Johannes Gutenberg University Mainz, Mainz, Germany; ^3^Faculty of Mathematics and Natural Sciences, Institute of Pharmaceutical and Medicinal Chemistry, Heinrich Heine University Düsseldorf, Düsseldorf, Germany

**Keywords:** Huntington’s disease, class IIa histone deacetylases, RNA stability, RNA-binding protein, polyglutamine protein

## Abstract

**Introduction:**

Huntington’s disease (HD) is a fatal inherited neurological disorder for which there are no curative treatments available. Patients suffer from progressive impairment in cognitive and motor functions. Transcriptional dysregulation is a major molecular disease mechanism of HD. Transcription is regulated by a variety of epigenetic marks, including acetylation of histone proteins. This acetylation is controlled by opposing activities of histone acetyltransferases and histone deacetylases (HDACs). Based on recent observations that inhibition of HDACs can ameliorate disease phenotypes in different model systems ranging from cell culture to yeast, *Drosophila* and mouse models, the development of HDAC inhibitors as therapeutics for HD is promising. Recently, class IIa HDAC enzymes (4, 5, 7, 9) and specifically HDAC 4, have been identified as potential targets for the treatment of HD.

**Methods:**

Here, we tested a set of novel class IIa HDAC inhibitors for their efficiency in two different HD models: an HD cell line model and a *Drosophila* model.

**Results:**

The selective class IIa HDAC inhibitor 1a led to a significant reduction of HTT aggregation and ameliorated the disease phenotype *in vivo*. The reduction in HTT aggregates was caused by reduced RNA levels in treated samples, due to decreased RNA stability.

**Discussion:**

Our data suggest a so far unknown mode of action of HDAC class IIa inhibitors by affecting *HTT* transcript levels.

## 1 Introduction

Huntington’s disease (HD) is an incurable neurodegenerative disorder characterized by progressive impairment in cognitive and motor functions. HD is caused by expansion of a polyglutamine-coding CAG repeat in exon 1 of the *huntingtin* (*HTT*) transcript. Individuals with a repeat expansion > 39 CAGs display near-complete disease penetrance. A pathological hallmark of HD is formation of aggregates containing mutant polyglutamine HTT protein in the patients’ brains. Although the underlying molecular pathogenesis leading to neuronal loss remains to be comprehensively explained, transcriptional dysregulation is a major pathogenic characteristic (reviewed in Jiang et al., 2023).

Interestingly, the transcriptional regulation by chromatin acetylation is connected to HD pathology (reviewed in Sharma and Taliyan, 2015). The acetylation of histones is regulated by opposing actions of histone acetyltransferases and histone deacetylases (HDACs). The HDAC protein family (HDACs 1–11 and class III sirtuins 1–7) is involved in transcriptional repression and chromatin condensation via the removal of acetyl groups from histones and other non-histone proteins. The HDAC family contains 11 zinc-dependent enzymes grouped into three classes (classes I, II, and IV). HDACs 1–3 and 8 are class I enzymes. Class II HDACs can be further subdivided into class IIa (HDACs 4, 5, 7, and 9) and class IIb (HDACs 6 and 10).

There is increasing evidence that HDAC inhibitors may represent a useful class of therapeutic agents for HD: For example, in transgenic mouse models of HD, the HDAC class IIa isoform HDAC4 associates with mutant HTT protein and colocalizes with cytoplasmic aggregates. Moreover, HDAC4 reduction delays aggregate formation, restores expression of brain-derived neurotrophic factor (BDNF), and ameliorates the disease phenotype (Mielcarek et al., 2013). In addition, prolonged treatment of an HD mouse model with vorinostat (or suberoylanilide hydroxamic acid: SAHA), which is an inhibitor of class I HDACs as well as the class IIb enzyme HDAC6, caused a decrease in HDAC4 and HDAC2 levels in the cortex and brain stem without affecting their transcript levels. This was accompanied by a decrease in aggregate load in the same brain regions as well as by restoration of *Bdnf* cortical transcript level (Mielcarek et al., 2011). In another study, oral administration of vorinostat in drinking water significantly improved motor impairment in an HD mouse model (Hockly et al., 2003). All these observations suggest that the development of HDAC inhibitors as therapeutics for HD is a promising approach.

Since broad-spectrum HDAC inhibitors have adverse side-effects in chronic dosing paradigms, which would be essential for the treatment of HD, our goal was to selectively target class IIa HDACs. This concept was based on a report describing that genetic suppression of the HDAC class IIa isoform HDAC4 in an HD mouse model ameliorates neurological phenotypes and extends lifespan (Mielcarek et al., 2013).

## 2 Methods

### 2.1 HDAC inhibitors

A mini-library of trifluoromethyloxadiazol (TFMO)-based selective HDAC class IIa inhibitors, recently described by the Kurz group (Asfaha et al., 2024; Bollmann et al., 2022), was screened to identify a lead compound for more comprehensive biological studies in HD.

TFMO-based selective HDAC class IIa inhibitors are characterized by a TFMO Zn-binding group, a phenyl linker, an amide connecting unit in the para position of the linker, and a basic tertiary amine cap group in one defined distance.

### 2.2 Filter retardation assay

HEK293 cells stably expressing *HTT* exon1 with 83 CAG repeats (Waelter et al., 2001) were treated with different HDAC inhibitors (1 μM) and incubated for 72 h at 37° C and 8% CO_2_. Filter retardation assays were performed as described elsewhere (Scherzinger et al., 1997). Samples were analyzed in triplicates. For quantification, band intensities were measured using iBright software (Invitrogen). Normalization was performed using the band intensity of ACTB on western blots of the same samples (anti-actin beta-HRP,1:1000 5% BSA in TBST; Cell Signaling Technology).

### 2.3 Immunofluorescence staining

HEK293 cells stably expressing HTT exon1 with 83 CAG repeats (Waelter et al., 2001) were seeded on poly-L-lysine coated coverslips. After 72 h, cells were washed with PEM buffer and fixed in 4% paraformaldehyde (in PEM for 10 min). Followed by washing with 1x PBS and permeabilization by incubation in 0.2% TritonX (in 1x PBS) for 10 min. The cells were washed again in 2 ml 1x PBS and blocked in 2% BSA (in 1x PBS) for 30–60 min. Primary antibody: Anti-Flag (DYKDDDDK AlexaFlour® 647, 1:100; ThermoFisher). Slides were embedded with 10 μl mounting medium + 0.5 μg/ml DAPI (SIGMA) and the cell covered side of the coverslips. Confocal microscopy was done using an LSM900 microscope (Zeiss). Image analysis was done using the ZEN software (Zeiss).

### 2.4 Actinomycin D timecourse

HEK293 cells stably expressing *HTT* exon1 with 83 CAG repeats (Waelter et al., 2001) were treated with the HDAC inhibitor 1a (f.c. 1 μM; stock 1 mM). After induction of *HTT* exon1 cells were incubated for 60 h. Then the actinomycin D timecourse was started. For this purpose, 1 μl actinomycin D (5 mg/ml; Merck) was added per ml medium and the cells were harvested after 0, 3, 6, 9, and 12 h. Total RNA was isolated using the Monarch® Total RNA Miniprep Kit (NEB). cDNA was synthesized using the TaqMan reverse transcription reagents kit (Applied Biosystems) and real-time PCR was carried out (Primer: GAPDH-for: CCACATCGCTCAGACACCAT, GAPDH-rev: AAATCCGTTGACTCCGACCTT; mutHTTexon1-Flag-for: CGCG GCCCCGAATT, mutHTTexon1-Flag-rev: TCTTTGTAGTCCAT GGTGGTTCA; HTTexon7-for: GTGGTAAGCCGCCTG; HTTexon7-rev: GAGCACATTTAGTAGCCAAC) using the qPCRBIO SyGreen Mix (PCRBiosystems).

### 2.5 *In vivo* treatment

*Drosophila melanogaster* lines GMR-Gal4 (Bloomington stock center No.8605) and P{UAS-HTT.ex1. Q97}3 (Bloomington stock center No.68417) were crossed. To prepare the experimental fly food instant medium (Formula 4–24, Carolina) supplemented with a little dry yeast was used. Inhibitor 1a was first dissolved in DMSO solution (10 mM) and then diluted with water and added to the fly food at a final concentration of 0.5 μM. Similarly diluted DMSO was used as a control. After hatching from the pupae, the flies were divided into males and females and frozen for 30 min at -80°C. The heads were then separated using tweezers under a microscope (Leica). For each batch, 20 heads per gender were pooled for filter retardation assay. The heads were homogenized with lysis buffer and pestles. Filter retardation assays were performed as described elsewhere (Scherzinger et al., 1997). Samples were analyzed in duplicates. The membrane was detected via anti-HTT antibody (1:1000, 2% BSA in TBST; Millipore) and FITC anti-mouse (1:1000, 1% BSA in TBST; Abcam) using iBright (Invitrogen). For quantification, signal intensities on the membranes were measured using the iBright analysis software (Invitrogen). Normalization of the filter retardation assay signals was performed using the band intensity of ACTB (anti-β-actin; 1:1000, 1% BSA in TBST; Cell Signaling technology; mouse anti-rabbit IgG-HRP, 1:1000, 1% BSA in TBST; Santa Cruz Biotechnology) western blots blotted onto nitrocellulose membranes (ThermoFisher).

### 2.6 RNA pull-down and western blots

HTT exon1 DNA (49 CAG) was PCR-amplified (Primer: HTT-Exon1 T7 for: CCAAGCTTCTAATACGACTCACTATAGGG AGAATGGCGGA CCCTGGAAAAGCTCATGAAGG, HTT-Exon1 rev: GGTCG GTGCAGCGGCT CCTCAGC). The forward primer was designed to add a T7 sequence. The PCR product was purified using the Hi Yield® Gel/PCR DNA Fragment Extraction Kit (Südlabor). *In vitro* transcription was performed using the RiboMAX Large-scale RNA production system-T7 (Promega) with the addition of 100 nM biotin-rUTP (ThermoFisher). The resulting RNAs were purified by phenol-chloroform extraction. 500 μg HEKT protein lysate was incubated with 200 pmol biotinylated RNA and 1% RNAse inhibitor, with and without addition of 1 μM inhibitor 1a for 1 h at RT. The samples were crosslinked (2 min, 200 mJ/cm^2^). 20 μl high-capacity streptavidin-agarose beads (ThermoFisher) were added to all samples and incubated rotating over night at 4°C. Subsequently, the high-capacity streptavidin-agarose beads were washed twice with 1 ml washing buffer (25 mM Tris, pH 7.4, 15 mM NaCl, 1% NP40, and 0.5% sodium deoxycholate) for 10 min while rotating. The RNA-bound proteins were analyzed by western blot. Western blots were done using semi-dry blotting on nitrocellulose-membranes. Signals were detected using the Class II HDAC Antibody Sampler Kit (#79891 Cell Signalling), the Acetyl-Histone Antibody Sampler Kit (#9933 Cell Signalling), anti-FUS/TLS (sc-44711, Santa Cruz) and anti-mouse HRP (Cell Signaling) antibodies.

### 2.7 RNA immunoprecipitation

HEK293 cells stably expressing *HTT* exon1 with 83 CAG repeats (Waelter et al., 2001) were treated with the HDAC inhibitor 1a (f.c. 1 μM; stock 1 mM). After induction of *HTT* exon1 cells were incubated for 72 h. Cells were harvested in PBS using a cell scraper and subjected to UV crosslink (150 mJ/cm^2^ x 10 min). The cell pellet was resuspended in 1 ml TKM Buffer (containing protease inhibitor and RNAse inhibitor). The lysates were precleared using protein g agarose (Roche). The pre-cleared lysates were subjected to immunoprecipitation using 2 μg of anti-FUS antibody (Santa Cruz, sc-47711) and 50 μl protein g agarose per sample following the manufacturer’s instructions. RNA was extracted from the immunoprecipitated FUS-complexes by phenol-chloroform extraction. cDNA synthesis and qRT-PCR were done as described above. An aliquot of the immunoprecipitated sample was analyzed on western blots detecting the FUS-protein to control the immunoprecipitated protein level. The Ct-values of the qRT-PCR were analyzed using a standard curve (serial dilution). The resulting data was then normalized to the signal intensity of the FUS protein (in %).

## 3 Results

### 3.1 Selection of novel class IIa HDAC inhibitors

The class IIa inhibitors were synthesized as reported previously (Asfaha et al., 2024; Bollmann et al., 2022). Based on their potent and selective class IIa HDAC inhibition and low cytotoxicity against human cells, a mini-library of TFMO-based class IIa selective HDAC inhibitors was selected to identify a potential lead compound for HD. The previously published IC_50_ values of the compounds at class IIa HDAC enzymes were: inhibitor 1c: HDAC4 12 nM, HDAC5 19 nM, HDAC7 46 nM; inhibitor 2b: HDAC4 114 nM, HDAC5 268 nM, HDAC7 284 nM; inhibitor 2c: HDAC4 141 nM, HDAC5 151 nM, HDAC7 263 nM (Asfaha et al., 2024; Bollmann et al., 2022). All inhibitors were substantially less effective on class I, class IIb and class IV HDACs (Asfaha et al., 2024). TFMO-based HDAC inhibitors feature a unique heterocyclic, non-chelating Zn-binding group (TFMO), providing significant advantages over hydroxamic acid-based HDAC inhibitors in terms of selectivity, toxicity, chemical reactivity, and polarity. Furthermore, TFMO-based HDAC inhibitors have demonstrated improved physicochemical properties essential for effective brain penetration (Stott et al., 2021).

### 3.2 Inhibitor 1a reduces HTT aggregates

**Figure 1:**
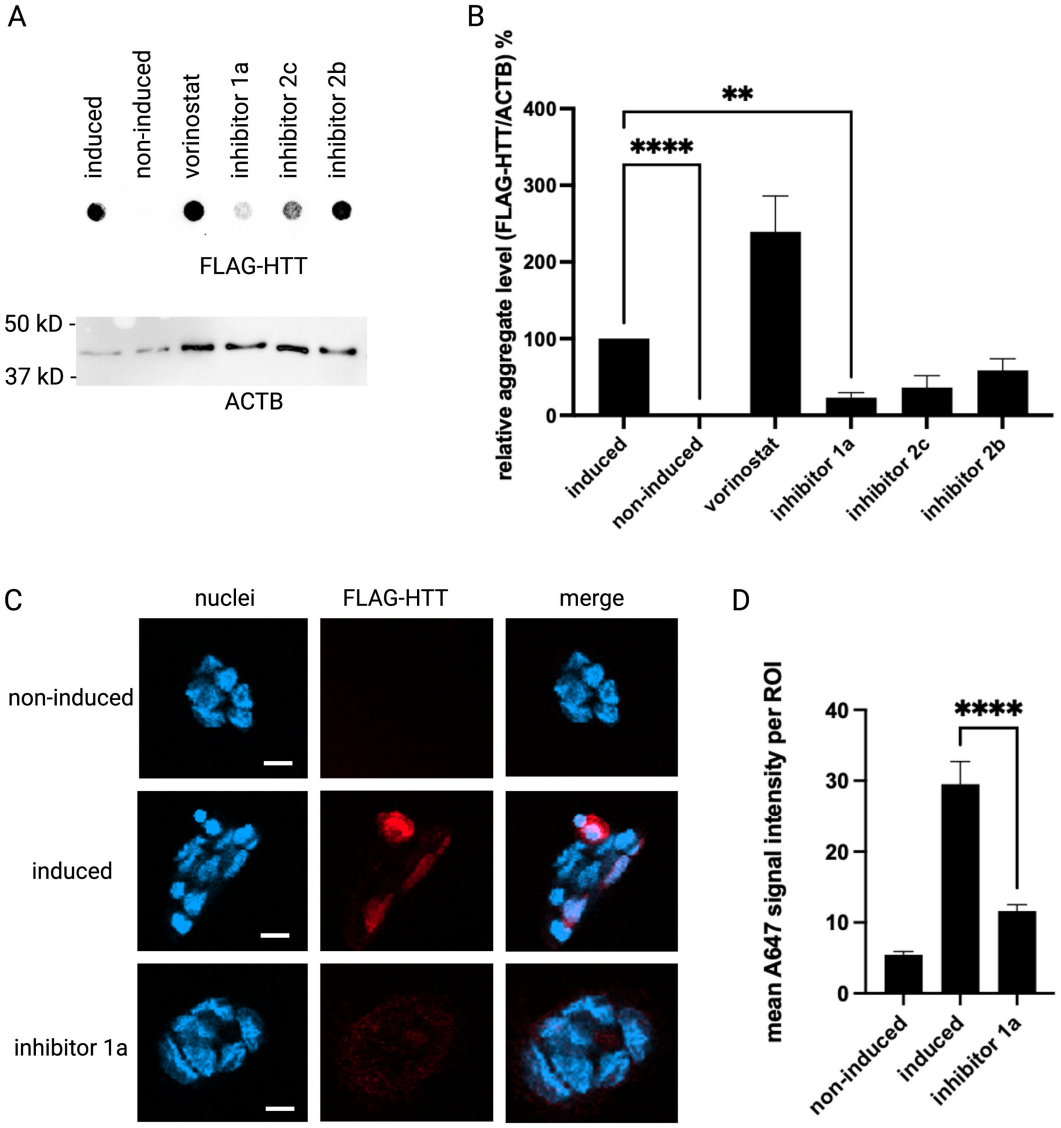
HDAC inhibitor 1a reduces the amount of aggregation-prone mutant HTT protein in HEKQ83 cells. **(A)** Upper panel: Filter retardation assay from cell lysates of HEK293 cells stably expressing mutant *HTT* exon1 (induced) in the absence or presence of different HDAC inhibitors. The cell line without induction of mutant *HTT* exon1 expression (non-induced) served as a control. Lower panel: As a loading control a western blot is shown on which ACTB was detected in the same samples. **(B)** Quantification of mutant HTT exon1 aggregate level (%) normalized to ACTB. Columns represent mean values +/-SEM. One-way ANOVA followed by a Dunnett test was performed to indicate significant efficiency. *n* = 4, p**** < 0.0001, p** < 0.005. **(C)** Immunofluorescence staining of cells expressing mutant *HTT* exon 1. Cells were immuno-stained for FLAG-tagged HTT exon 1 (red, AlexaFlour® 647) and the nucleus (blue, DAPI). **(D)** Quantification of AlexaFlour® 647 from cells as described in **(C)**. Columns represent mean fluorescent signal intensity per ROI +/− SEM (*n* = 33 ROIs). p**** < 0.0001 (unpaired t test).

To assess the efficiency of the set of novel HDAC class IIa inhibitors described above in an HD cell model, we used a well-established HEK293 model stably expressing mutant *HTT* exon1 with 83 CAG repeats under a tet-off inducible promoter (Waelter et al., 2001) and treated these cells with the different class IIa HDAC inhibitors. The amount of aggregated mutant HTT protein was measured in a filter retardation assay. Out of the inhibitors tested, inhibitor 1a caused a significant decrease in the relative aggregate level (Figures 1A,B). We selected this compound for further experiments. An immunofluorescence staining of the same cell line treated with or without inhibitor 1a confirmed the significant reduction of the mutant HTT protein upon treatment (Figures 1C,D).

### 3.3 Inhibitor 1a reduces *HTT* RNA level and stability

**Figure 2:**
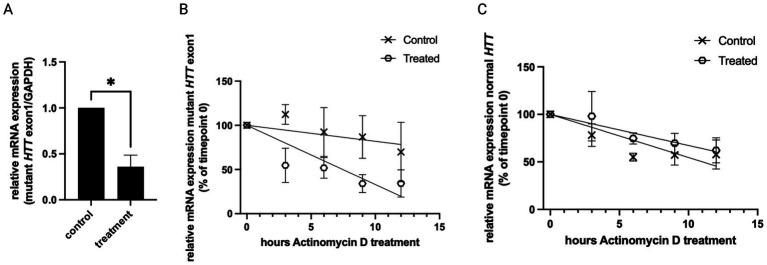
Mutant *HTT* exon1 RNA stability is affected by inhibitor 1a. **(A)** qRT-PCR analysis of mutant *HTT* exon1 expression normalized to *GAPDH* in HEK293 cells stably expressing mutant *HTT* exon1 with 83 CAG repeats in the absence (control) or presence of inhibitor 1a (treatment). Columns represent mean values +/− SEM. Welch’s t test was performed to indicate significant efficiency. *n* = 4, p* < 0.05. **(B)** Mutant *HTT* exon1 RNA stability decreases upon inhibitor 1a treatment. RNA was quantified in a qRT-PCR (normalized to *GAPDH*). Relative mutant *HTT* exon1 RNA abundance was measured in an actinomycin D timecourse experiment. Graph represents RNA decay in control and inhibitor 1a treated cells after actinomycin D treatment. Different mRNA levels are shown as percent of timepoint 0; mean values of *n* = 3 +/− SEM. The resulting data were analyzed by two-way ANOVA. The factor treatment with inhibitor 1a significantly (*p* = 0.0474) affects RNA stability. **(C)** Endogenous normal *HTT* RNA stability does not change upon inhibitor 1a treatment. RNA was quantified in a qRT-PCR (normalized to *GAPDH*). Relative normal *HTT* RNA abundance was measured in an actinomycin D timecourse experiment as described in **(B)**. While the factor time affects RNA stability (*p* = 0.016), the factor treatment with inhibitor 1a does not cause a significant difference (*p* = 0.3706).

The above-mentioned decrease in HTT protein and aggregates may be explained in different ways: it may be caused by an increase in protein degradation, or by a decrease in protein synthesis, e.g., by a decrease in RNA transcript encoding the protein. To test if inhibitor 1a influences the mutant *HTT* exon1 transcript level, we treated the above-mentioned HD cell model with or without inhibitor 1a and measured the RNA level by qRT-PCR. Interestingly, treatment with inhibitor 1a significantly reduced the mutant *HTT* exon1 RNA level (Figure 2A), indicating that the observed reduction in mutant *HTT* occurs on the transcript level.

To test if this reduced level of mutant *HTT* exon1 RNA can be explained by destabilization of the RNA upon inhibitor 1a treatment, we measured the half-life time of the RNA transcript in the presence or absence of inhibitor 1a. Measurements of decay rates of mRNAs can be done by analyzing the mRNA abundance following transcription inhibition. Here, we used actinomycin D, which is widely used in mRNA stability assays. It intercalates into DNA, preventing the unwinding of the DNA double-helix, thereby inhibiting the DNA-dependent RNA polymerase activity. Following actinomycin D treatment for different periods of time, we measured the mRNA level by qRT-PCR. In Figure 2B the decay rates of mutant *HTT* exon1 in cells with or without inhibitor 1a treatment are visualized. We observed a significant increase in decay of mutant *HTT* exon1 RNA in treated cells. This suggests that inhibitor 1a affects RNA stability of the mutant *HTT* exon1 transcript. Interestingly, there was no difference in decay rates of the endogenous normal *HTT* RNA in the same cells (Figure 2C).

**Figure 3:**
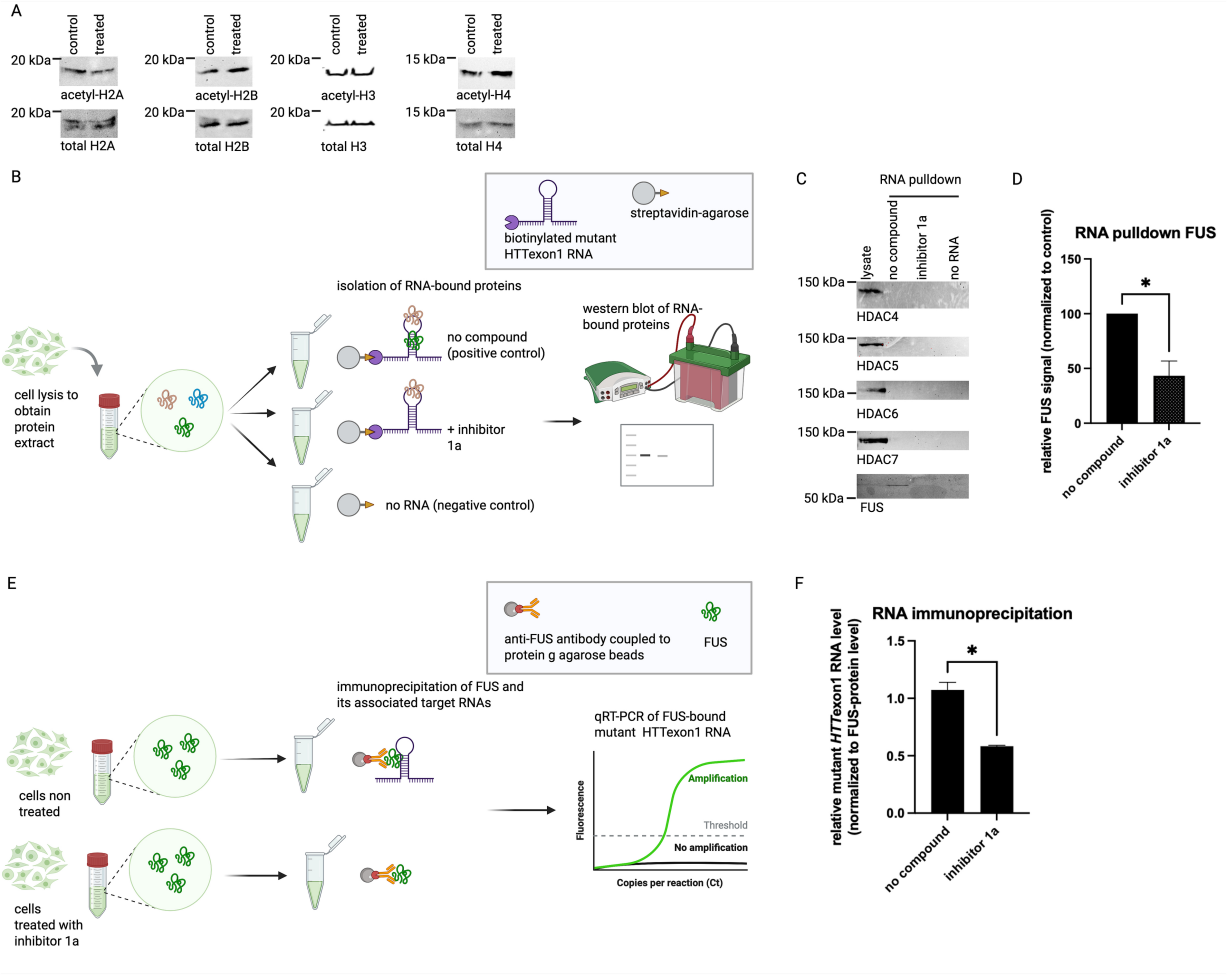
Inhibitor 1a treatment does not affect histone-acetylation. **(A)** HeLa cells were treated with or without 1 μM inhibitor 1a for 24 h. Protein extracts of these cells were analyzed on western blots detecting acetylated histones H2A (Lys 5), H2B (Lys 5), H3 (Lys 9) and H4 (Lys 8). Total histones H2A, H2B, H3 and H4 were detected in the same samples as loading controls. A representative image of *n* = 3 experiments is shown. Quantitative analysis of the western blots revealed no statistically significant differences; *p*-values (two-tailed t test): H2A *p* = 0.4639, H2B *p* = 0.3274, H3 *p* = 0.3129 and H4 *p* = 0.2998. **(B)** Schematic of RNA-pulldown assay to assess RNA-protein-interaction. Biotinylated *HTT*-RNA (49 CAG repeats) was immobilized on streptavidin-agarose beads. RNA-coated beads were incubated with protein lysates to allow RNA-protein binding either in the absence (positive control) or presence of 1 μM inhibitor 1a overnight at 4°C. As a negative control (no RNA), a sample without biotin-labeled RNA was used. The RNA-bound proteins were eluted and analyzed on a western blot. **(C)** RNA-pulldown assay to test the binding of class II HDACs to *HTT*-RNA (49 CAG repeats). While the HDACs were well detectable in total cell lysates, no efficient co-purification with mutant *HTT* RNA was detectable. In contrast, the RNA-binding protein FUS was detected to bind to *HTT* RNA in the absence of inhibitor 1a, while treatment with inhibitor 1a abolished this binding. **(D)** Quantification of *n* = 4 RNA pulldown assays detecting FUS. Columns represent mean values +/− SEM of relative FUS-protein level, normalized to the no compound control. * *p* = 0.0249. **(E)** Schematic of RNA-immunoprecipitation to assess RNA-protein-interaction. HEK293 cells stably expressing mutant *HTT* exon1 were treated with or without inhibitor 1a. The FUS-protein was immunoprecipitated using a FUS-antibody coated onto protein g agarose beads. RNA was isolated from the immunoprecipitated samples and FUS-bound *HTT*exon1 RNA was analyzed using qRT-PCR. **(F)** Relative mutant *HTT*exon1 RNA level in the immunoprecipitated samples described in **(E)** normalized to the FUS-protein level in the immunoprecipitated samples. Columns represent mean values +/− SEM. Two-tailed t test was performed to indicate significant efficiency. *n* = 3, * *p* = 0.0161. Schematics were created using biorender.com.

Generally, HDACs are involved in transcriptional repression and chromatin condensation via the deacetylation of histones. Therefore, after inhibition of HDACs an increase in expression of the HDAC’s target genes is generally expected. Since we observe a different effect, namely the destabilization of mutant *HTT* RNA, histone acetylation is unlikely to explain the effect of inhibitor 1a. In line with this notion, treatment with inhibitor 1a did not increase the acetylation status of histones H2A, H2B, H3 or H4 (Figure 3A). Thus, another mechanism must drive the seen effect. To assess the possibility that class II a HDACs may directly bind to mutant *HTT* RNA and thereby affect its stability, we performed RNA-protein pulldown assays. In brief, *in-vitro* transcribed mutant *HTT* exon1 RNA was immobilized on agarose-beads. The RNA-coated beads were incubated with protein extracts to allow RNA-protein binding in the presence or absence of inhibitor 1a (Figure 3B). The presence of class IIa HDACs in the fraction of RNA-bound proteins was then analyzed on western blots. No class IIa HDAC could be detected in these samples, suggesting that the HDACs do not strongly attach to mutant *HTT* RNA (Figure 3C). Thus, we assumed that class IIa HDACs may affect mutant *HTT* RNA stability indirectly by regulating an RNA-binding protein that binds to and regulates mutant *HTT* RNA. Our previous studies identified RNA-binding proteins that attach to *HTT* exon1 RNA (Schilling et al., 2019). We searched this published set of proteins for known interaction partners of class IIa HDACs and found the FUS protein as an interesting candidate. Therefore, we tested if inhibitor 1a may affect the binding of mutant *HTT* RNA to FUS. Interestingly, we observed a substantial decrease in the amount of FUS protein bound to the mutant *HTT* RNA in the samples treated with inhibitor 1a (Figure 3C). This suggests that inhibitor 1a reduces binding of HDAC-interacting proteins like FUS to the *HTT* RNA.

### 3.4 Inhibitor 1a reduces HTT aggregates *in vivo* and rescues disease phenotype (*Drosophila melanogaster*)

**Figure 4:**
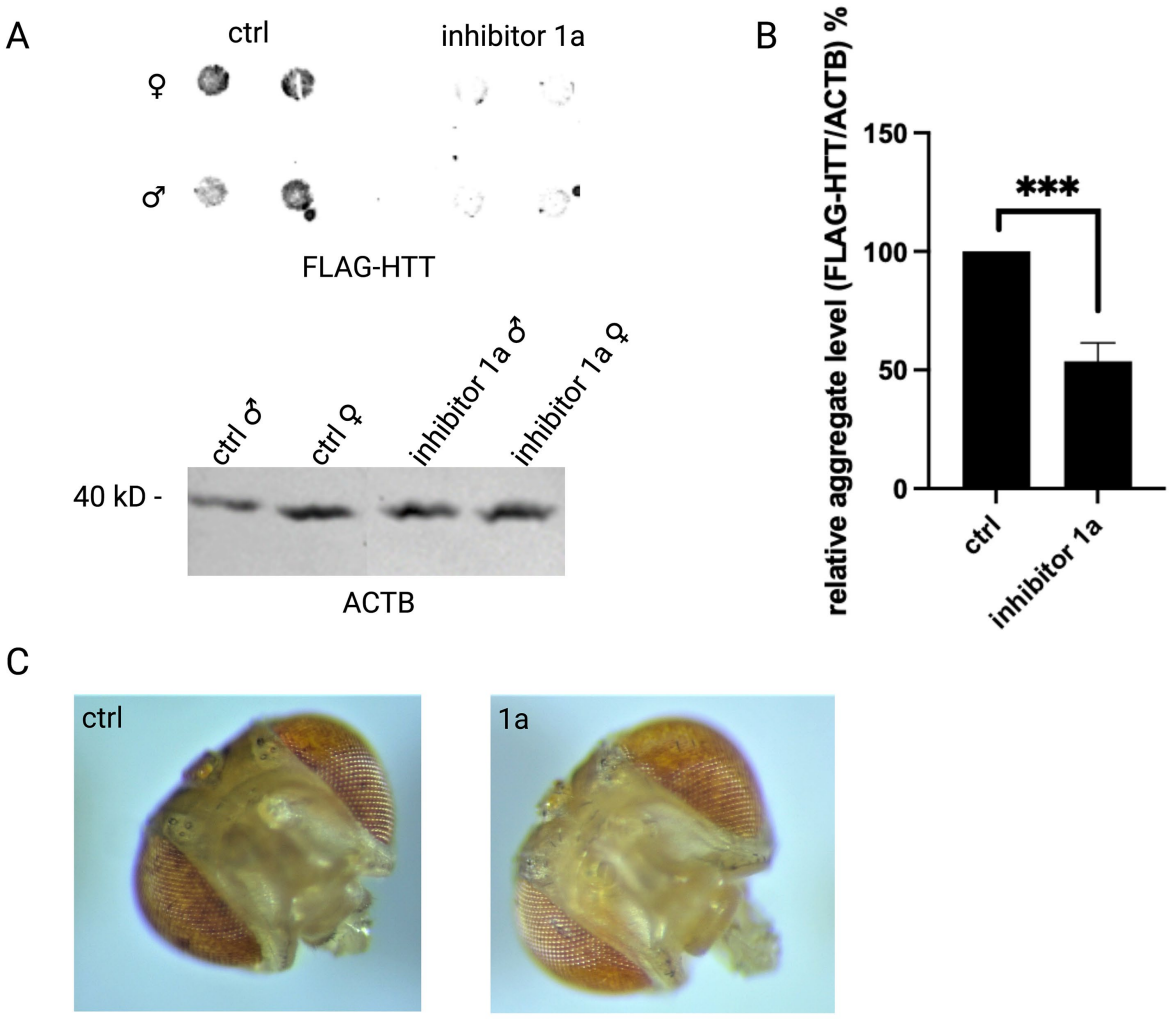
Inhibitor 1a reduces mutant HTT protein level and disease phenotype *in vivo*. **(A)** Filter retardation assay (left) was performed using lysates from an HD *Drosophila* model expressing *HTT* exon1 with 97 CAG repeats treated with inhibitor 1a. *Drosophila melanogaster* without treatment were used as control (ctrl). The experiments were performed in 4 replicates, each replicate contained a pool of 20 flies (in sum 80 flies were analyzed per condition). Upper panel: Filter retardation assay detecting HTT. Lower panel: As a loading control a western blot is shown on which ACTB was detected in the same samples. **(B)** Quantification of mutant HTT exon1 aggregate level (%) normalized to ACTB. Columns represent mean values +/− SEM. Welch’s t-test was performed to indicate significant efficiency. p** < 0.01. **(C)** Image of *Drosophila melanogaster* eyes with (inhibitor 1a) and without (ctrl) inhibitor 1a treatment.

In the next set of experiments, we wanted to test the effect of inhibitor 1a *in vivo*. Therefore, we used an HD *Drosophila* model expressing *HTT* exon1 with 97 CAG repeats. The fly larvae were fed with fly medium with or without inhibitor 1a. After hatching, the flies were analyzed by different methods. The aggregate level was assessed via filter retardation assay (Figure 4A). In line with our results from the HD cell line model, inhibitor 1a treatment also significantly reduced the aggregate level *in vivo* (Figure 4B). Moreover, a typical eye phenotype of this *Drosophila* model was assessed: expression of *HTT* exon1 with 97 CAG repeats leads to death of pigment cells and a rough eye phenotype in control flies, which was partially suppressed by inhibitor 1a (Figure 4C).

## 4 Discussion

Epigenetic factors can profoundly influence gene expression and, in turn, influence disease pathways. Thus, epigenetic drugs, such as HDAC inhibitors, are promising drug candidates, although their exact mechanisms of action remain elusive. Here, we investigated the effect of a novel class IIa HDAC inhibitor in HD models and found that this inhibitor ameliorates disease phenotypes both *in cellulo* in an HD cell model as well as *in vivo* in a *Drosophila* model.

Different molecular mechanisms are affected in HD and contribute to disease development, some of which are connected to histone acetylation. For example, the transcriptional deregulation, a well-known phenomenon in HD brain (Hodges et al., 2006; Kuhn et al., 2007; Seredenina and Luthi-Carter, 2012; Vashishtha et al., 2013; Yildirim et al., 2019; Malla et al., 2021), could be explained by decreased histone acetylation. Indeed, decreased histone acetylation leading to transcriptional dysregulation was observed in a HD mouse model (McFarland et al., 2012). One molecular explanation of such decrease in histone acetylation in HD models is that mutant HTT protein inhibits acetyltransferase activity by sequestering activators of histone acetylation (Steffan et al., 2001). Another study suggests that HDAC inhibition affects regulation of genes in protein degradation pathways like the ubiquitin–proteasomal system and autophagy. Thereby HDAC inhibition may affect accumulation, stability, and clearance of important disease-related proteins, like HTT (Jia et al., 2012). In line, an HDAC inhibitor that preferentially targets HDAC1 and HDAC3 ameliorates the HD phenotype in different HD models like transgenic mice (Jia et al., 2012). Moreover, mutant HTT protein recruits HDAC4 to aggregates (Mielcarek et al., 2013), which may account for differences in histone acetylation. Interestingly, HDAC4 reduction delays cytoplasmic aggregate formation, restores BDNF expression and ameliorates the disease phenotype in HD models. However, HDAC4 reduction did neither affect global transcriptional deregulation nor did it modulate nuclear HTT aggregation (Mielcarek et al., 2013). This may hint to a so far unknown function of HDAC4 or its target proteins that is not directly reflected by transcriptional deregulation. Another disease mechanism in HD that has been connected to HDACs is the phenomenon of somatic CAG repeat expansion that drives the rate of pathogenic pathways. Interestingly, the genetic knockout of *HDAC2* or *HDAC3* in medium-spiny striatal neurons moderately attenuated CAG expansion in an HD mouse model (Kovalenko et al., 2020). In our study we found that a class IIa HDAC inhibitor decreases HTT aggregate load in HD models by destabilizing the *HTT* RNA transcript, representing another so far unknown mechanism that connects HDACs and mutant *HTT*.

Generally, HDACs are involved in transcriptional repression and chromatin condensation via the removal of acetyl groups from histones and nonhistone proteins. Thus, if we treat our model with an HDAC inhibitor, we may expect an increase in expression of the HDAC’s target genes. However, we detected a destabilization of the mutant *HTT* transcript upon HDAC inhibition, pointing to a so far unknown mode of action of HDACs on the mutant *HTT* RNA. Inhibitor 1a is a selective class II a HDAC inhibitor (Asfaha et al., 2024). It inhibits class II a HDAC at low IC50 values (HDAC 4 0.012 μM, HDAC 5 0.019 μM, HDAC7 0.046 μM), while the IC50 is much higher for class I HDACs (HDAC 1 4.02 μM, HDAC 2 6.13 μM, HDAC 3 3.82 μM, HDAC 8 4.26 μM) as well as class IIb HDACs (HDAC 6 5.79 μM) or class IV HDACs (HDAC 11 66.0 μM) (Asfaha et al., 2024). Interestingly, class IIa HDACs have unique features that distinguish them from other HDACs. They have low deacetylase activity, and they contain an N-terminal domain that interacts with transcription factors and recruits them to their target genes. This N-terminal domain further enables the phospho-dependent transport of the class IIa HDACs from the nucleus to the cytoplasm. Unphosphorylated class IIa HDACs are located in the nucleus, where they interact with transcription factors and repress their target genes. Upon phosphorylation, class IIa HDACs translocate to the cytoplasm and de-repress their target genes (reviewed in Parra, 2014). Thus, upon inhibition of class IIa HDACs by inhibitor 1a one may expect an increased expression of its target genes. However, we observed the opposite effect, namely a destabilization of mutant *HTT* RNA after treatment with inhibitor 1a indicating that class IIa HDACs do not repress mutant *HTT* expression. Since many RNA-binding proteins are involved in the regulation of RNA-stability, one possible explanation for the seen effect may be that inhibitor 1a affects the binding of RNA-binding proteins to mutant *HTT* RNA. Our previous studies showed that *HTT* exon1 RNA recruits different proteins including FUS (Schilling et al., 2019). Interestingly, treatment with our HDAC inhibitor 1a decreases the binding between mutant *HTT* RNA and FUS. Thus, we speculate that FUS binds to and stabilizes mutant *HTT* RNA in an HDAC-dependent manner, which can be blocked by HDAC inhibition. FUS is an RNA-binding protein that has been linked to diverse neurodegenerative diseases including amyotrophic lateral sclerosis (ALS). In ALS FUS regulates repeat associated non-ATG translation (RAN translation) through modulating the G-quadruplex structure of GGGGCC repeat RNA in C9orf72-linked (Fujino et al., 2023). Interestingly, HDAC6 inhibition reverses axonal transport defects in motor neurons derived from FUS-ALS patients (Guo et al., 2017; Tejido et al., 2021), suggesting a functional connection between class II HDAC inhibition and FUS, which is in line with our data. Functionally, inhibition of class II HDACs could promote acetylation of the FUS protein thereby affecting its RNA-binding. Alternatively, class II HDACs may act as scaffolds influencing the RNA-binding of FUS.

Our data suggest that FUS is connected to HD. This finding is supported by previous studies showing that FUS is a disease-modifier in HD: FUS co-aggregates with mutant HTT and heterozygous knockout of FUS worsens the phenotypes of HD mice (Kino et al., 2016). Several of the above-mentioned studies endorse the use of HDAC inhibitors to treat neurodegenerative diseases like HD. With respect to adverse side-effects of broad-spectrum HDAC inhibitors, the development of novel HDAC inhibitors with improved selectivity, potency, safety, and brain penetration will greatly advance the therapeutic potential HDAC inhibitors for chronic administration in diseases like HD. A recent study describes the design, synthesis, and *in vitro* characterization of highly isoform-selective HDAC4 protein degraders (Macabuag et al., 2022). Clinical trials using Selisistat, a selective of inhibitor of the deacetylating enzyme SirT1, in patients suffering from HD showed that this drug is safe and well tolerated. However, no beneficial effect on motor, cognitive or functional outcome measures were detectable, as this most likely requires longer treatment periods (Sussmuth et al., 2015). We used here a selective class IIa HDAC inhibitor, inhibitor 1a. Inhibitor 1a represents a promising lead compound for the treatment of HD, improving disease outcomes in both an HD cell model and *in vivo*. This is attributed to its potent and selective class IIa HDAC inhibition, which reduces HTT aggregates and exerts a previously unknown effect on mutant *HTT* RNA stability. To further assess the potential of inhibitor 1a as a lead for HD drug development, pharmacokinetic and toxicological studies are required in the future.

In conclusion, inhibition of class IIa HDACs, especially HDAC4, ameliorates the disease phenotype in HD models. Our data support that selective class IIa HDAC inhibitors are attractive candidates to treat HD. Recent optimizations of these inhibitors improved their selectivity, potency, and brain penetration advance their therapeutic potential. Here we show that the selective class IIa HDAC inhibitor 1a ameliorates the disease outcome in both an HD cell model and *in vivo*. Moreover, our data point to a so far unknown mode of action of HDACs on mutant *HTT* RNA stability. Future studies shall address the use of this inhibitor in preclinical/clinical application.

## Data Availability

The original contributions presented in the study are included in the article/supplementary material, further inquiries can be directed to the corresponding author.
